# Trends in Characteristics and Outcomes of Hospital Inpatients Undergoing Coronary Revascularization in the United States, 2003-2016

**DOI:** 10.1001/jamanetworkopen.2019.21326

**Published:** 2020-02-14

**Authors:** Mohamad Alkhouli, Fahad Alqahtani, Ankur Kalra, Sameer Gafoor, Mohamed Alhajji, Mohammed Alreshidan, David R. Holmes, Amir Lerman

**Affiliations:** 1 Department of Cardiology, Mayo Clinic School of Medicine, Rochester, Minnesota; 2 Division of Cardiology, Department of Medicine, University of Kentucky, Lexington; 3 Department of Cardiovascular Medicine, Cleveland Clinic, Cleveland, Ohio; 4 Swedish Heart and Vascular Institute, Seattle, Washington; 5 King Fahad Medical City, Riyadh, Saudi Arabia

## Abstract

**Importance:**

Data on the contemporary changes in risk profile and outcomes of patients undergoing percutaneous coronary intervention (PCI) or coronary bypass grafting (CABG) are limited.

**Objective:**

To assess the contemporary trends in the characteristics and outcomes of patients undergoing PCI or CABG in the United States.

**Design, Setting, and Participants:**

This retrospective cohort study used a national inpatient claims-based database to identify patients undergoing PCI or CABG from January 1, 2003, to December 31, 2016. Data analysis was performed from July 15 to October 4, 2019.

**Main Outcomes and Measures:**

Demographic characteristics, prevalence of risk factors, and clinical presentation divided into 3 eras (2003-2007, 2008-2012, and 2013-2016) and in-hospital mortality of PCI and CABG stratified by clinical indication.

**Results:**

A total of 12 062 081 revascularization hospitalizations were identified: 8 687 338 PCIs (72.0%; mean [SD] patient age, 66.0 [10.8] years; 66.2% male) and 3 374 743 CABGs (28.0%; mean [SD] patient age, 64.5 [12.4] years; 72.1% male). The annual PCI volume decreased from 366 to 180 per 100 000 US adults and the annual CABG volume from 159 to 82 per 100 000 US adults. A temporal increase in the proportions of older, male, nonwhite, and lower-income patients and in the prevalence of atherosclerotic and nonatherosclerotic risk factors was found in both groups. The percentage of revascularization for myocardial infarction (MI) increased in the PCI group (22.8% to 53.1%) and in the CABG group (19.5% to 28.2%). Risk-adjusted mortality increased slightly after PCI for ST-segment elevation MI (4.9% to 5.3%; *P* < .001 for trend) and unstable angina or stable ischemic heart disease (0.8% to 1.0%; *P* < .001 for trend) but remained stable after PCI for non–ST-segment elevation MI (1.6% to 1.6%; *P* = .18 for trend). Risk-adjusted CABG morality markedly decreased in patients with MI (5.6% to 3.4% for all CABG and 4.8% to 3.0% for isolated CABG) and in those without MI (2.8% to 1.7% for all CABG and 2.1% to 1.2% for isolated CABG) (*P* < .001 for all).

**Conclusions and Relevance:**

Significant changes were found in the characteristics of hospital inpatients undergoing PCI and CABG in the United States between 2003 and 2016. Risk-adjusted mortality decreased significantly after CABG but not after PCI across all clinical indications.

## Key Points

**Question:**

What are the contemporary trends in the characteristics and outcomes of patients undergoing coronary revascularization in hospitals in the United States?

**Findings:**

In this cohort study of patients undergoing percutaneous coronary intervention and coronary bypass grafting in hospitals in the United States from 2003 to 2016, risk-adjusted mortality temporally decreased significantly after coronary bypass grafting but not after percutaneous coronary intervention across all clinical indications.

**Meaning:**

This study revealed changes in the clinical profile of hospital inpatients referred for coronary revascularization and in the temporal trends of risk-adjusted mortality of percutaneous coronary intervention and coronary bypass grafting in the United States from 2003 to 2016.

## Teaser

This cohort study assesses the contemporary trends in the characteristics and outcomes of patients undergoing percutaneous coronary intervention and coronary bypass grafting.

## Introduction

Coronary artery revascularization has affected millions of patients with coronary artery disease (CAD) worldwide. Both surgical and percutaneous revascularization strategies have evolved from experimental stages to routine procedures that can safely tackle complex coronary anatomic features and high-risk patients.^1,2,3^ Several studies^4,5,6,7^ have documented a significant decrease in coronary artery bypass grafting (CABG) operations after the emergence of percutaneous coronary interventions (PCI) in the 1990s. However, the annual volumes of both PCI and CABG decreased significantly in more recent years possibly because of advances in medical therapy, the emergence of data questioning the benefit of PCI in stable CAD, and the increasing implementation of appropriate use criteria.^8,9,10,11^ Whether these temporal changes in procedural volume were associated with changes in the risk profiles of patients referred for percutaneous or surgical coronary revascularization and the outcomes of these procedures remain unknown. This study used a nationwide, representative sample hospital inpatients in the United States to assess the temporal changes in baseline characteristics of patients undergoing PCI or CABG and crude and risk-adjusted in-hospital mortality after PCI or CABG stratified by clinical indication.

## Methods

### Study Data

We conducted a retrospective cohort study using the Nationwide Inpatient Sample (NIS) database to derive patient-relevant information from January 1, 2003, to December 31, 2016. Data analysis was performed from July 15 to October 4, 2019. The West Virginia University Institutional Review Board exempted the study from board approval and waived the requirement for informed consent because the NIS is a publicly available deidentified database. This study followed the Strengthening the Reporting of Observational Studies in Epidemiology (STROBE) reporting guideline.^12^

The NIS is part of the Healthcare Cost and Utilization Project (HCUP), sponsored by the Agency for Healthcare Research and Quality, and is the largest publicly available all-payer claims-based database in the United States. The database contains hospital inpatient stays derived from billing data submitted by hospitals to statewide data organizations across the United States. These data include clinical and resource use information typically available from discharge abstracts. Researchers and policy makers use the NIS to make national estimates of health care utilization, access, charges, quality, and outcomes. The NIS sampling frame includes data from 47 statewide data organizations, covering more than 97% of the US population. The annual sample encompasses approximately 8 million discharges, which represent 20% of inpatient hospitalizations across different hospital types and geographic regions. The national estimates of the entire US hospitalized population are calculated using a standardized sampling and weighting method provided by the HCUP. The NIS has been used extensively to assess national trends in the utilization, disparities, and outcomes of coronary artery interventions.^13,14,15,16,17,18,19^

### Study Population

Patients aged 18 years or older who underwent PCI or CABG between 2003 and 2016 were identified using *International Classification of Diseases, Ninth Revision, Clinical Modification (ICD-9-CM)* and *International Statistical Classification of Diseases and Related Health Problems, Tenth Revision (ICD-10)* codes (eTable 1 in the Supplement). We further classified PCIs into those performed for ST-segment elevation myocardial infarction (STEMI), non–ST-segment elevation myocardial infarction (NSTEMI), or unstable angina or stable ischemic heart disease (UA-SIHD). Given the rarity of CABG performed in the context of STEMI, we classified CABG operations into 2 groups: CABG in the context of acute myocardial infarction (AMI) and CABG performed for UA-SIHD.

### Study Outcomes

Our study investigated trends in clinical risk profile among hospital inpatients undergoing PCI and CABG divided into 3 eras (2003-2007, 2008-2012, and 2013-2016). These eras were selected to provide relatively equal periods and to illustrate the global and not the year-to-year change in baseline characteristics among patients undergoing coronary revascularization. The study also investigated trends in the crude and adjusted in-hospital mortality associated with PCI and CABG, stratified by clinical indication.

### Statistical Analysis

Weighted data were used for all statistical analyses. Descriptive statistics are presented as numbers with percentages for categorical variables. Means (SDs) are used to report continuous measures. To evaluate changes in baseline characteristics by calendar year, we used the Mantel-Haenszel test of trend for categorical variables and linear regression for continuous variables. To assess whether in-hospital mortality improved over time, multivariable logistic regression models were constructed to estimate the odds ratios and 95% CIs. To directly estimate rate ratios, a modified Poisson regression approach was used that included a robust variance estimate in the models.^20^ Calendar year was included as a categorical variable, with 2003 as the reference year. All the multivariable regression models used in risk-adjusted estimates were fitted with generalized estimating equations to account for clustering of outcomes within hospitals. Adjusted risk ratios and *P* values for trend were determined with a model evaluating calendar year as a continuous variable. Variables included in the regression models included demographic characteristics (age, sex, and race/ethnicity), socioeconomic factors (primary expected payer and median household income), Elixhauser comorbidity index score, and clinically relevant comorbidities (eTable 2 in the Supplement). The trend weight files were merged onto the original NIS files by year and hospital identification number. For years before 2012, the trend weight was used to create national estimates for trend analysis. For 2012 and after, no trend weight was needed, and the regular discharge weight was used, consistent with the redesigned NIS trend analysis.^21^

Statistical analysis was performed accounting for data changes in trend analysis and avoiding use of nonspecific secondary diagnosis codes to infer in-hospital events. Methodologic standards in research using the NIS were met as recommended.^22^ A 2-sided *P* < .05 was considered to be statistically significant. All statistical analyses were performed with SPSS software, version 24 (IBM Corp).

## Results

**Figure 1.**
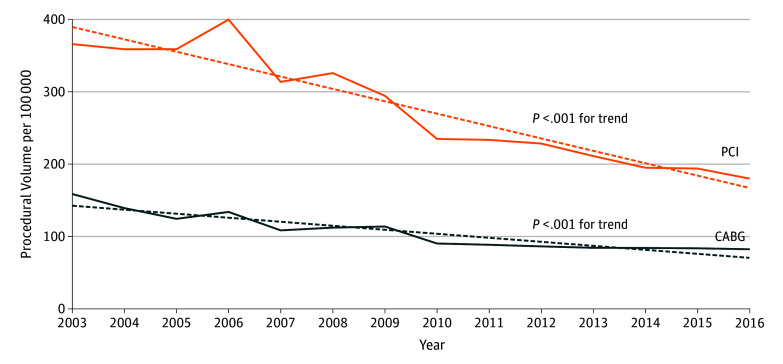
Temporal Trend in the Annual Rate of Percutaneous and Surgical Coronary Revascularization per 100 000 US Adults Dashed line indicates the mean trend and solid line the year-to-year trend. CABG indicates coronary artery bypass grafting; PCI, percutaneous coronary intervention.

A total of 12 062 081 revascularization hospitalizations were identified: 8 687 338 PCIs (72.0%; mean [SD] patient age, 66.0 [10.8] years; 66.2% male) and 3 374 743 CABGs (28.0%; mean [SD] patient age, 64.5 [12.4] years; 72.1% male). The annual PCI volume decreased from 777 780 in 2003 to 440 505 in 2016 (eTable 3 in the Supplement). This volume corresponded to a decrease in the PCI rate from 366 to 180 per 100 000 US adults between 2003 and 2016 (Figure 1). Similarly, the annual CABG volume decreased from 337 444 in 2003 to 201 840 in 2016, corresponding to a decrease in the CABG rate from 159 to 82 per 100 000 US adults between 2003 and 2016 (Figure 1 and eTable 3 in the Supplement). Significant temporal changes occurred in the demographic characteristics, socioeconomic status, prevalence of risk factors, and clinical presentations of patients undergoing PCI and CABG and in the characteristics of the procedures.

In the PCI group, a temporal increase occurred in the proportions of older and male patients, nonwhite patients, and patients with lower socioeconomic status. There was also a significant increase in the prevalence of atherosclerotic and nonatherosclerotic risk factors (Table 1). The proportion of patients with an Elixhauser comorbidity index score of 3 or greater increased from 24.7% in 2003 to 2007 to 52.3% in 2012 to 2016. The proportion of women among all patients undergoing PCI decreased from 34.0% in 2003 to 2006 to 32.8% in 2012 to 2016 (*P* < .001). The proportion of women among all patients undergoing CABG decreased from 29.0% in 2003 to 2006 to 26.0% in 2012 to 2016 (*P* < .001). The percentage of PCI for AMI among all PCIs increased from 22.8% in 2003 to 2007 to 53.1% in 2012 to 2016. The characteristics of PCIs changed as well. Patients who underwent PCI between 2012 and 2016 (vs those who underwent PCI between 2003 and 2007) had fewer multivessel PCIs (16.2% vs 17.9%) and used bare metal stents less (15.5% vs 27.4%) but had more PCIs for chronic total occlusion (3.4% vs 0.1%) and cardiogenic shock (5.0% vs 1.8%) and had greater use of intravascular ultrasonography and/or fractional flow reserve (9.2% vs 2.5%) and circulatory support devices (4.6% vs 2.5%) (*P* < .001 for all).

**Table 1.** Temporal Changes in Baseline Characteristics of Patients Undergoing PCI

Characteristic	No. (%) of Patients			*P* Value
	2003-2007 (n = 3 903 880)	2008-2012 (n = 3 014 784)	2013-2016 (n = 1 877 560)	
Age group, y				
18-44	247 642 (6.3)	188 443 (6.3)	117 660 (6.3)	<.001
45-64	1 780 008 (45.6)	1 398 869 (46.4)	860 580 (45.9)	
65-85	1 760 169 (45.1)	1 308 598 (43.4)	808 450 (43.1)	
>85	115 866 (3.0)	117 442 (3.9)	89 395 (4.8)	
Male	2 577 536 (66.0)	2 001 981 (66.4)	1 262 295 (67.2)	<.001
Race/ethnicity				
White	2 253 800 (81.1)	1 990 655 (77.6)	1 349 530 (76.2)	<.001
Black	191 753 (6.9)	218 667 (8.5)	167 815 (9.5)	
Hispanic	181 206 (6.5)	177 861 (6.9)	137 035 (7.7)	
Insurance status				
Medicare or Medicaid	2 180 979 (55.9)	1 722 609 (57.1)	1 143 600 (60.9)	<.001
Private insurance	1 457 469 (37.3)	1 010 461 (33.5)	564 490 (30.1)	
Self-pay, no charge, or other	266 432 (6.8)	281 713 (9.3)	169 470 (9.1)	
Median household income percentile				
25th or less	942 566 (24.7)	812 354 (27.6)	544 030 (29.6)	<.001
26th to 50th	991 652 (26.0)	804 655 (27.3)	501 995 (27.3)	
51st to 75th	965 878 (25.3)	722 146 (24.5)	438 885 (23.9)	
76th to 100th	911 243 (23.9)	609 413 (20.7)	354 625 (19.3)	
Clinical risk profile				
Hypertension	2 489 275 (63.7)	2 168 609 (71.9)	1 439 130 (76.6)	<.001
Hyperlipidemia	2 309 175 (59.1)	2 080 045 (69.0)	1 332 050 (70.9)	<.001
Diabetes	1 155 633 (29.6)	1 048 592 (34.8)	741 985 (39.5)	<.001
Peripheral vascular disease	351 605 (9.0)	330 253 (11.0)	207 630 (11.1)	<.001
Carotid artery disease	51 115 (1.3)	58 899 (2.0)	39 750 (2.1)	<.001
Atrial fibrillation	35 1003 (9.0)	329 513 (10.9)	265 080 (14.1)	<.001
Tobacco use	695 873 (17.8)	701 355 (23.3)	485 475 (25.9)	<.001
Chronic kidney disease	207 215 (5.3)	355 170 (11.8)	307 465 (16.4)	<.001
Chronic lung disease	532 688 (13.6)	487 872 (16.2)	331 270 (17.6)	<.001
Liver cirrhosis	3552 (0.1)	7674 (0.3)	8445 (0.4)	<.001
Anemia	288 790 (7.4)	361 795 (12)	266 935 (14.2)	<.001
Prior ICD or pacemaker	108 197 (2.8)	118 047 (3.9)	82 125 (4.4)	<.001
Prior stroke	6790 (0.2)	141 195 (4.7)	120 925 (6.4)	<.001
Prior sternotomy	31 1232 (8.0)	233 310 (7.7)	159 790 (8.5)	<.001
Elixhauser comorbidity index score				
0	607 173 (15.7)	272 375 (9.2)	102 740 (5.6)	<.001
1 or 2	2 300 813 (59.6)	1 486 866 (50.1)	774 055 (42.2)	
≥3	952 083 (24.7)	1 210 893 (40.8)	959 615 (52.3)	
Clinical presentation				
STEMI	281 148 (7.2)	276 299 (9.2)	262 858 (14.0)	<.001
NSTEMI	610 382 (15.6)	74 0476 (24.5)	734 126 (39.1)	<.001
UA-SIHD	3 013 350 (77.2)	1 998 009 (66.3)	899 351 (47.9)	<.001
PCI characteristics				
Multivessel PCI	700 094 (17.9)	558 706 (18.5)	304 010 (16.2)	<.001
IVUS or FFR use	98 279 (2.5)	209 119 (6.9)	172 580 (9.2)	<.001
Chronic total occlusion	3981 (0.1)	27 624 (0.9)	63 815 (3.4)	<.001
Bare metal stent use	1 069 908 (27.4)	796 417 (26.4)	290 290 (15.5)	<.001
Cardiogenic shock	68 815 (1.8)	96 811 (3.2)	94 290 (5.0)	<.001
Mechanical circulatory support	99 421 (2.5)	111 459 (3.7)	86 095 (4.6)	<.001

Abbreviations: FFR, functional flow reserve; ICD, internal cardioverter defibrillator; IVUS, intravascular ultrasonography; NSTEMI, non–ST-segment elevation myocardial infarction; PCI, percutaneous coronary intervention; SIHD, stable ischemic heart disease; STEMI, ST-segment elevation myocardial infarction; UA, unstable angina.

The CABG group also had a temporal increase in the proportion of male, elderly, and nonwhite patients and patients with lower socioeconomic status. Similar to what was observed in the PCI cohort, the prevalence of clinical risk factors increased significantly over time (Table 2). The proportion of patients with an Elixhauser comorbidity index score of 3 or greater increased from 29.8% in 2003 to 2007 to 52.2% in 2012 to 2016. The indications for CABG and surgical techniques also evolved over time. Compared with the 2003 to 2006 era, in 2012 to 2016, CABG was performed in a greater proportion of patients with AMI (28.2% vs 19.5%) and cardiogenic shock (6.1% vs 2.8%); however, these CABGs were more likely to be limited to 1 to 2 vessels (65.3% vs 55.6%), use arterial conduits (87% vs 82.2%), use double mammary conduits (3.7% vs 3.0%), or be isolated (86.9% vs 84.5%) but were less likely to use off-pump techniques (24.0% vs 19.3%) (*P* < .001 for all). Perioperative intra-aortic balloon pump use decreased from 9.7% to 8.9% (*P* < .001).

**Table 2.** Temporal Changes in Baseline Characteristics of Patients Undergoing CABG

Characteristic	No. (%) of Patients			*P* Value
	2003-2007 (n = 3 903 880)	2008-2012 (n = 3 014 784)	2013-2016 (n = 1 877 560)	
Age group, y				
18-44	50 698 (3.5)	37 283 (3.3)	24 410 (3.0)	<.001
45-64	613 065 (42.5)	484 470 (43.1)	339 640 (42.1)	
65-85	756 028 (52.4)	582 286 (51.8)	428 670 (53.2)	
>85	22 717 (1.6)	21 064 (1.9)	13 550 (1.7)	
Male	1 024 799 (71.0)	812 237 (72.2)	596 675 (74.0)	<.001
Race/ethnicity				
White	841 708 (81.6)	769 556 (79.9)	600 295 (79.3)	<.001
Black	61 374 (5.9)	64 731 (6.7)	51 815 (6.8)	
Hispanic	69 860 (6.8)	64 328 (6.7)	55 190 (7.3)	
Insurance status				
Medicare or Medicaid	856 805 (59.4)	676 874 (60.1)	512 505 (63.5)	<.001
Private insurance	499 217 (34.6)	366 737 (32.6)	242 480 (30.1)	
Self-pay, no charge, or other	86 756 (6.0)	81 839 (7.3)	51 530 (6.4)	
Median household income percentile				
25th or less	351 838 (25.0)	303 151 (27.6)	220 635 (27.9)	<.001
26th to 50th	375 422 (26.7)	308 057 (28.0)	216 725 (27.4)	
51st to 75th	356 692 (25.4)	269 452 (24.5)	195 470 (24.7)	
76th to 100th	322 637 (22.9)	219 297 (19.9)	157 195 (19.9)	
Clinical risk profile				
Hypertension	949 134 (65.8)	852 859 (75.8)	664 750 (82.4)	<.001
Hyperlipidemia	762 116 (52.8)	756 775 (67.2)	609 885 (75.6)	<.001
Diabetes	482 495 (33.4)	447 328 (39.7)	387 215 (48.0)	<.001
Peripheral vascular disease	482 495 (33.4)	447 328 (39.7)	387 209 (48.0)	<.001
Carotid artery disease	50 951 (3.5)	65 716 (5.8)	54945 (6.8)	<.001
Atrial fibrillation	416 162 (28.8)	340 256 (30.2)	279 635 (34.7)	<.001
Tobacco use	211 054 (14.6)	207 930 (18.5)	153 245 (19.0)	<.001
Chronic kidney disease	108 848 (7.5)	157 725 (14.0)	143 860 (17.8)	<.001
Chronic lung disease	309 968 (21.5)	247 771 (22.0)	171 295 (21.2)	<.001
Liver cirrhosis	2333 (0.2)	5085 (0.5)	4335 (0.5)	<.001
Anemia	301 410 (20.9)	357 145 (31.7)	239 865 (29.7)	<.001
Prior ICD or pacemaker	20 121 (1.4)	24 899 (2.2)	21 260 (2.6)	<.001
Prior stroke	31 741 (2.2)	59 132 (5.3)	54 510 (6.8)	<.001
Prior sternotomy	22 846 (1.6)	21 013 (1.9)	18 900 (2.3)	<.001
Elixhauser comorbidity index score				
0	172 966 (12.0)	102 427 (9.1)	45 315 (5.7)	<.001
1 or 2	839 434 (21.5)	558 991 (49.8)	336 500 (42.1)	
≥3	430 377 (11.0)	461 778 (41.1)	417 785 (52.2)	
Clinical presentation				
AMI	283 124 (19.6)	253 889 (22.6)	227 550 (28.2)	<.001
UA-SIHD	1 159 654 (80.4)	871 561 (77.4)	578 965 (71.8)	<.001
CABG characteristics				
Isolated CABG	1 254 402 (86.9)	947 936 (84.2)	681 535 (84.5)	<.001
1- to 2-vessel CABG	802 131 (55.6)	690 089 (61.3)	527 080 (65.3)	<.001
≥3-Vessel CABG	640 647 (44.4)	435 361 (38.7)	279 435 (34.7)	<.001
Off-pump CABG	346 184 (24.0)	263 261 (23.4)	155 755 (19.3)	<.001
Double IMAs	42 658 (3.0)	39 590 (3.5)	29 745 (3.7)	<.001
Any arterial conduit	1 187 603 (82.3)	960 166 (85.3)	701 395 (87.0)	<.001
Cardiogenic shock	39 859 (2.8)	55 835 (5.0)	49 125 (6.1)	<.001
Intra-aortic balloon pump use	129 223 (9.0)	109 317 (9.7)	71 635 (8.9)	<.001

Abbreviations: AMI, acute myocardial infarction; CABG, coronary artery bypass grafting; ICD, internal cardioverter defibrillator; IMA, internal mammary artery; SIHD, stable ischemic heart disease; UA, unstable angina.

**Figure 2.**
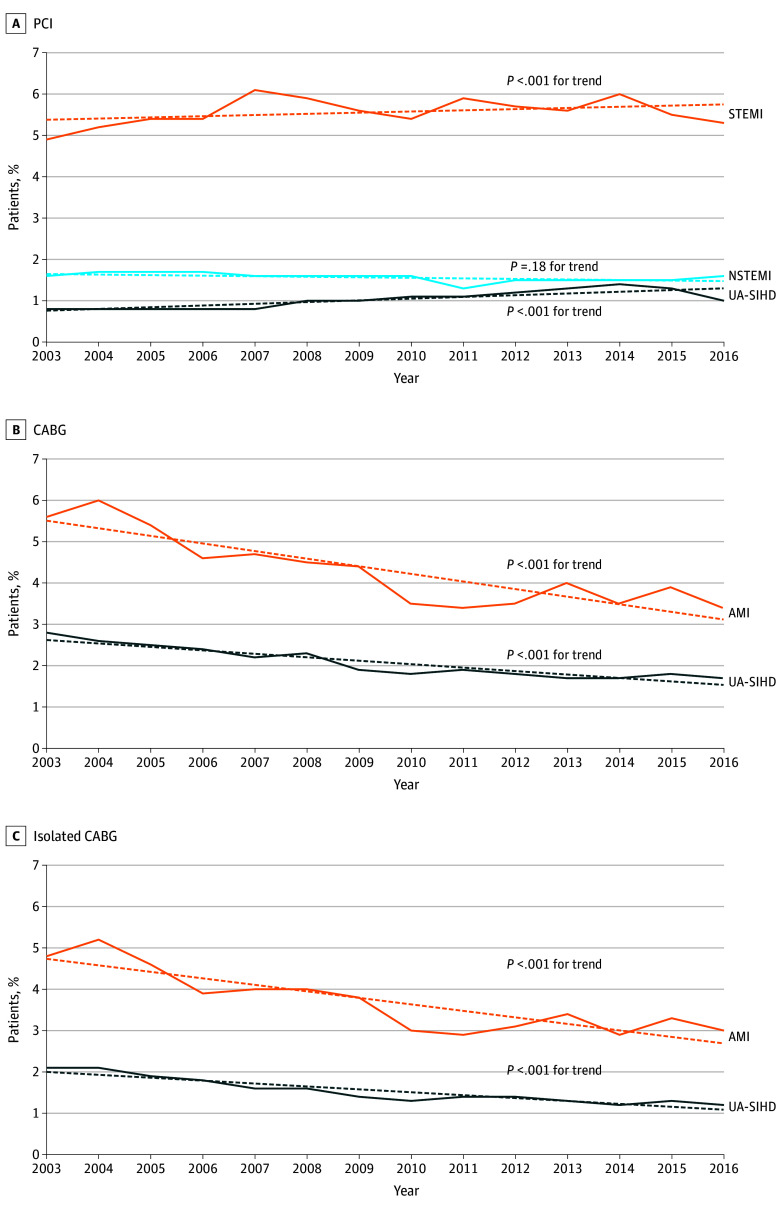
Temporal Trend in the Risk-Adjusted In-Hospital Mortality With Coronary Revascularization Stratified by Clinical Indication Dashed line indicates the mean trend and solid line the year-to-year trend. AMI indicates acute myocardial infarction; CABG, coronary artery bypass grafting; PCI, percutaneous coronary intervention; NSTEMI, non–ST-segment elevation myocardial infarction; SIHD, stable ischemic heart disease; STEMI, ST-segment elevation myocardial infarction; and UA; unstable angina.

In-hospital mortality after PCI increased between 2003 and 2016 (eTable 4 in the Supplement). However, after risk adjustment for patient- and hospital-level characteristics, in-hospital mortality only modestly increased after PCI for STEMI (4.9% to 5.3%; *P* < .001 for trend) or UA-SIHD (0.8% to 1.0%; *P* < .001) but remained stable after PCI for NSTEMI (1.6% to 1.6%; *P* = .18) (Figure 2A). In contrast, in-hospital mortality after isolated or combined CABG decreased significantly between 2003 and 2016 (eTable 5 in the Supplement). This temporal improvement in CABG mortality persisted after risk adjustment in both patients undergoing CABG in the context of AMI (5.6% to 3.4%; *P* < .001 for trend) or for UA-SIHD (2.8% to 1.7%; *P* < .001 for trend) (Figure 2B). Similar trends were observed when the analysis was limited to patients who underwent isolated CABG (Figure 2C and eTable 6 in the Supplement) or when we excluded patients who underwent both PCI and CABG during the same admission (eTable 7 in the Supplement). Length of stay after revascularization decreased across all groups, revascularization methods, and indications except among patients who underwent PCI for UA-SIHD (eTable 8 in the Supplement).

## Discussion

This study documents 3 major findings. First, a decrease in the number of percutaneous and surgical coronary revascularization procedures conducted among hospital inpatients in the United States was found between 2003 and 2016. Second, significant changes were found in the demographic characteristics, socioeconomic status, risk profile, and clinical presentation of hospital inpatients undergoing coronary revascularization over time, as well as a significant change in the characteristics of the revascularization procedures. Third, a temporal decrease was found in in-hospital mortality after CABG but not after PCI across various indications.

Several studies^4,5,6,9,11,14,23^ have found a decrease in coronary revascularization procedures in the United States in the past 2 decades. However, most of these studies^4,5,6,9,11,14,23^ were not contemporaneous, included only certain subsets of patients (eg, patients with Medicare insurance or AMI), or examined trends of surgical or percutaneous revascularization procedures. Although our primary objective was to assess the temporal changes among hospital inpatient risk profiles, procedural characteristics, and procedural mortality, the current study, to our knowledge, provides the most up-to-date nationwide analysis of the annual trends in coronary interventions conducted in hospitals. We documented a 40% decrease in CABG volume and a 43% decrease in PCI volume between 2003 and 2016. However, these downward trends appeared to stabilize at approximately 200 000 CABG procedures annually and 450 000 PCIs annually, with CABG volume reaching a steady level earlier than PCI volume (2010 vs 2014). Albeit speculative, reasons for these downward trends in the earlier years of our study may include the change in the management of stable CAD after the publication of landmark clinical trials reporting the effectiveness of medical management of stable CAD,^8,24,25^ the implementation of appropriate use criteria, and the improved efficacy of CAD preventive measures.^8,10,14,26^ The increasing proportion of patients with AMI among all patients undergoing PCI (22.8% to 53.1%) and CABG (19.6% to 28.2%) over time further supports this notion.

This study also found a temporal change in the demographic characteristics, socioeconomic status, and clinical risk profiles of hospital inpatients undergoing PCI or CABG and an evolution of the characteristics of these procedures. There was a modest increase in the number of elderly patients undergoing PCI or CABG but a more notable increase in the proportion of racial/ethnic minority patients and those with lower household income over time. Although this finding may reflect a change in the total population demographic characteristics and socioeconomic status during the same period, it may also partially reflect better penetration of coronary interventions to underserved populations.^27^ With regard to sex-related disparities in revascularization, not only did women remained underrepresented (approximately one-third overall) but also their proportion among all patients undergoing revascularization continued to decrease in both the CABG cohort (29.0% in 2003-2006 to 26.0% in 2012-2016, *P* < .001) and the PCI cohort (34.0% in 2003-2006 to 32.8% in 2012-2016, *P* < .001). Reasons for this disparity are likely multifaceted and deserve further investigations.

There was a marked increase in the prevalence of clinical risk factors among hospital inpatients undergoing revascularization over time, which was reflected by the doubling of the proportion of patients with an Elixhauser comorbidity index score of 3 or greater between 2003 and 2016 in both the PCI and the CABG cohorts. This increase was global for atherosclerotic risk factors (eg, hypertension, hyperlipidemia, and diabetes), nonatherosclerotic risk factors (eg, lung, renal, and liver disease), and concomitant noncoronary atherosclerosis (eg, carotid stenosis and vascular disease). This finding may represent an increase in the prevalence of certain risk factors in the total US population,^28,29^ the tendency to offer revascularization to sicker populations,^30^ the shift in risk resulting from performing fewer revascularization procedures in patients with stable CAD, or the database included in this study, which does not include PCIs conducted in outpatient settings, or a mixture of these factors. These findings have important implications for prerevascularization risk assessment and postrevascularization medical management. For example, the increasing prevalence of atrial fibrillation and anemia among hospital inpatients undergoing PCI may pose a challenge for a post-PCI antithrombotic and antiplatelet medical regimen.

The changes in hospital inpatient presentation and clinical risk profile were also accompanied by changes in coronary revascularization techniques over time. In the PCI cohort, there was an increasing uptake of intravascular ultrasonography and fractional flow reserve measurement and a downward trend in the use of bare metal stents. There was also an increasing representation of higher-risk patients (eg, cardiogenic shock and chronic total occlusion) but fewer multivessel PCIs. In the CABG cohort, there were fewer multivessel (>2) CABGs and off-pump CABGs but greater use of arterial conduits over time. These trends likely reflect the effect of the emerging data that suggest the incremental benefit of certain techniques or devices (eg, fractional flow reserve, drug-eluting stents, and arterial conduits) and the limited value of others (eg, off-pump bypass).^31,32,33,34,35,36,37,38^

We hypothesized that the temporal decrease in PCI and CABG volume, as well as the accompanying changes in hospital inpatient risk profile and procedural characteristics, might have been associated with a change in procedural mortality over time. We thus evaluated crude and risk-adjusted rates of in-hospital mortality of both procedures stratified by indication. We found that crude and risk-adjusted CABG mortality decreased significantly over time despite the substantial decrease in annual volume and the increasing prevalence of key comorbidities. Reasons for this trend may include changes in surgical techniques, the wider adoption of quality improvement, the changes in case mix and patient selection in light of the advances in PCI techniques, and public reporting of surgical outcomes, as well as the limitations of the NIS database.^39,40,41^

Contemporary data on the trends in PCI mortality are limited to subanalysis of specific PCI indications or certain subgroups of hospital inpatients. In a large study^42^ from the National Cardiovascular Data Registry CathPCI Registry, risk-adjusted in-hospital mortality of primary PCI for STEMI increased from 4.7% in 2005 to 5.3% in 2011 (*P* = .06). In another analysis^43^ from the same CathPCI registry, in-hospital mortality after PCI for cardiogenic shock increased over time. Goel et al^19^ found that in-hospital mortality after PCI in nonagenarians remained stable between 2003 and 2014 in the context of STEMI or NSTEMI but increased in the context of UA-SIHD.

To our knowledge, our study provides the largest contemporary analysis of the temporal trends of PCI mortality among hospital inpatients overall. In this analysis, we found that in-hospital mortality after PCI did not improve over time. These findings may seem counterintuitive because of the advances in PCI tools, technique, and quality (eg, drug-eluting stents, radial access, mechanical circulatory support, and door-to-balloon time); however, other factors could have offset the assumed mortality benefits of these tools and techniques (eg, decreased operator experience and inadequate adjustment for patient risk in our study’s database). These assumptions deserve further elaboration. The association between operator volume and outcomes after coronary revascularization has been both well-established historically and reconfirmed in contemporary analyses.^13,44,45^ Although the decrease in operator experience because of the decreasing volume of revascularization procedures applies to both CABG and PCI, we speculate that its association with outcomes might be greater with PCI because of the larger number of PCI operators. Similarly, the lack of granular anatomic, laboratory, and procedural data in the NIS may have influenced the robustness of our risk adjustment. Although this lack of data applies to both the PCI and CABG groups, it is possible that the addition of such data to the logistic regression models could have affected the PCI group more than the CABG group. For example, the complexity of coronary lesions (eg, bifurcation and calcification) may affect PCI outcomes more than CABG outcomes. Nonetheless, in light of these data, more studies are needed to identify effective strategies to further optimize PCI outcomes.

### Limitations

This study has limitations. First, the NIS is an administrative database that collects data for billing purposes. Thus, it is subject to undercoding, overcoding, or erroneous coding. However, coding of major procedures is the main method of obtaining reimbursement, and thus systematic inaccuracy in coding for PCI and CABG is unlikely. In addition, the NIS database has been used extensively to examine PCI and CABG trends and outcomes. Epstein et al^14^ validated the accuracy of the national estimation of annual volume with NIS by reporting a mean difference of 0.2% in quarterly PCI counts between Medicare claims and the NIS. Second, angiographic findings, laboratory data, characteristics of the PCI or CABG culprit vessel(s), access site, and perioperative medications are not available in NIS. Thus, the association of these unmeasured confounders with postrevascularization outcomes cannot be assessed. Third, the NIS allows detailed assessment of in-hospital outcomes but does not track patients after discharge or account for procedures conducted among outpatients. Therefore, long-term outcomes after PCI or CABG could not be investigated with this database. Despite these limitations, the NIS affords the unique opportunity to comprehensively assess the national trends in the utilization and outcomes of both percutaneous and surgical revascularization procedures in the United States during a 14-year period.

## Conclusions

There were considerable changes in the demographic characteristics, risk profile, and clinical presentation of patients undergoing PCI and CABG in hospitals that accompanied the substantial decrease in the annual volume of both procedures in the United states between 2003 and 2016. Risk-adjusted in-hospital mortality decreased over time after CABG but not after PCI across various clinical indications.

## Notes

Supplement. **eTable 1.** ICD-CM Codes Used to Identify the Study’s Cohort **eTable 2.** Variables Included in the Regression Models **eTable 3.** Temporal Trends in the Volume of PCI and CABG (Total Numbers and Rates Per 100.000 US Adults) **eTable 4.** Temporal Trends of Crude (unadjusted) and Risk-Adjusted In-Hospital Mortality Rates Following PCI **eTable 5.** Temporal Trends of Crude (unadjusted) and Risk-Adjusted In-Hospital Mortality Rates Following CABG **eTable 6.** Temporal Trends of Crude (unadjusted) and Risk-Adjusted In-Hospital Mortality Following Isolated CABG **eTable 7.** Temporal Trends in Unadjusted In-Hospital Mortality Among Patients Undergoing PCI, CABG, or PCI and CABG During the Same Hospitalization **eTable 8.** Temporal Trends of Length of Stay Following PCI and CABG
