# Clarification That Study Database Included Hospital Inpatients

**DOI:** 10.1001/jamanetworkopen.2020.6536

**Published:** 2020-04-28

In the Original Investigation titled, “Trends in Characteristics and Outcomes of Hospital Inpatients Undergoing Coronary Revascularization in the United States, 2003-2016,” published on February 14, 2020, in *JAMA Network Open*,^1^ the authors reported a retrospective cohort study using the Nationwide Inpatient Sample (NIS) administrative database. However, it was not clear that the study did not include those who received percutaneous coronary interventions in outpatient settings. This article and the related Invited Commentary^2^ have been corrected online to clarify that the study only included hospital inpatients.
