# Supplementary material for: Trends in Characteristics and Outcomes of Hospital Inpatients Undergoing Coronary Revascularization in the United States, 2003-2016
Source: JAMA Netw Open. 2020 Feb 14;3(2):e1921326. doi: 10.1001/jamanetworkopen.2019.21326 (PMC12507464; doi:10.1001/jamanetworkopen.2019.21326)
Supplement: Supplement. — eTable 1. ICD-CM Codes Used to Identify the Study’s Cohort eTable 2. Variables Included in the Regression Models eTable 3. Temporal Trends in the Volume of PCI and CABG (Total Numbers and Rates Per 100.000 US Adults) eTable 4. Temporal Trends of Crude (unadjusted) and Risk-Adjusted In-Hospital Mortality Rates Following PCI eTable 5. Temporal Trends of Crude (unadjusted) and Risk-Adjusted In-Hospital Mortality Rates Following CABG eTable 6. Temporal Trends of Crude (unadjusted) and Risk-Adjusted In-Hospital Mortality Following Isolated CABG eTable 7. Temporal Trends in Unadjusted In-Hospital Mortality Among Patients Undergoing PCI, CABG, or PCI and CABG During the Same Hospitalization eTable 8. Temporal Trends of Length of Stay Following PCI and CABG [file jamanetwopen-e1921326-s001.pdf]

Supplementary Online Content

Alkhouli M, Alqahtani F, Kalra A, et al. Trends in characteristics and outcomes of patients undergoing coronary revascularization in the United States, 2003-2016. *JAMA Netw Open*. 2020;3(2):e1921326. doi:10.1001/jamanetworkopen.2019.21326

eTable 1. ICD-CM Codes Used to Identify the Study's Cohort

eTable 2. Variables Included in the Regression Models

eTable 3. Temporal Trends in the Volume of PCI and CABG (Total Numbers and Rates Per 100.000 US Adults)

eTable 4. Temporal Trends of Crude (unadjusted) and Risk-Adjusted In-Hospital Mortality Rates Following PCI

eTable 5. Temporal Trends of Crude (unadjusted) and Risk-Adjusted In-Hospital Mortality Rates Following CABG

eTable 6. Temporal Trends of Crude (unadjusted) and Risk-Adjusted In-Hospital Mortality Following Isolated CABG

eTable 7. Temporal Trends in Unadjusted In-Hospital Mortality Among Patients Undergoing PCI, CABG, or PCI and CABG During the Same Hospitalization

eTable 8. Temporal Trends of Length of Stay Following PCI and CABG

This supplementary material has been provided by the authors to give readers additional information about their work.

© 2020 Alkhouli M et al. *JAMA Network Open.*

Variable	Source	Code(s)
**Percutaneous Coronary Intervention**	ICD-9-CM	00.66, 36.01, 36.02, 36.05, 36.06, 36.07, 17.55
	ICD-10-CM	0270346, 027034Z, 02703D6, 02703DZ, 02703Z6, 02703ZZ, 0270446, 027044Z, 02704D6, 02704DZ
**Acute Myocardial Infarction**		
STEMI	ICD-9-CM	410.00, 410.01, 410.10, 410.11, 410.20, 410.21, 410.30, 410.31, 410.40, 410.41, 410.50, 410.51, 410.60, 410.61, 410.80, 410.81, 410.90, 410.91
	ICD-10-CM	I21.01, I21.02, I21.09, I21.11<br>I21.19, I21.21, I21.29, I21.3, I22.0, I22.1, I22.2, I22.8, I22.9
NSTEMI		
	ICD-9-CM	410.70, 410.71, 411.1
**Coronary Artery Bypass Grafting**	ICD-10-CM	I21.4, I22.2
	ICD-9-CM	3610, 3611, 3612, 3613, 3614, 3615, 3616, 3617, 3619
	ICD-10-CM	02130KW, 02130Z3, 02130Z8, 02130Z9, 02130ZC, 02130ZF, 02130K8, 02130K9, 02130KC, 02130KF, 02130A9, 02130AC, 02130AF, 02130AW 02130J3, 02130J8 02130J9, 02130JC 02130JF, 02130JW 02130K3, 02120Z8, 02120Z9, 02120ZC, 02120ZF, 0213093, 0213098, 0213099, 021309C, 021309F, 021309W, 02130A3, 02130A8, 02120AW, 02120J3, 02120J8, 02120J9, 02120JC, 02120JF, 02120JW, 02120K3, 02120K8 02120K9, 02120KC 02120KF, 02120KW, 02120Z3, 02110Z9, 02110ZC, 02110ZF 0212093, 0212098, 0212099, 021209C, 021209F, 021209W, 02120A3, 02120A8, 02120A9, 02120AC 02120AF, 02110J3, 02110J8, 02110J9, 02110JC, 02110JF, 02110JW, 02110K3, 02110K8, 02110K9, 02110KC, 02110KF, 02110KW, 02110Z3, 02110Z8, 02100ZC 02100ZF 0211093 0211098, 0211099, 021109C, 021109F, 021109W, 02110A3, 02110A8, 02110A9, 02110AC, 02110AF, 02110AW, 02100J3, 02100K9, 02100KC, 02100KF, 02100KW, 02100Z3, 02100Z8, 02100Z9,0210093, 0210099, 021009C, 021009F, 021009W, 02100A3, 02100A8, 02100A9, 02100AC, 02100AF, 02100AW

eTable 1. ICD-CM Codes Used to Identify the Study's Cohort

STEMI, ST-elevation myocardial infarction; NSTEMI, non-ST-elevation acute coronary syndromes; ICD-CM, International Classification of Diseases

© 2020 Alkhouli M et al. *JAMA Network Open.*

**Demographics/Socioeconomics**<br>Age<br>Gender<br>Insurance Status<br>Median Household Income<br><br>**Clinical Risk Profile**<br>Hypertension<br>Hyperlipidemia<br>Diabetes Mellitus<br>Peripheral Vascular Disease<br>Carotid Artery Disease<br>Atrial Fibrillation<br>Tobacco Use<br>Chronic Kidney Disease<br>Chronic Lung Disease<br>Liver Cirrhosis<br>Anemia<br>Prior ICD/Pacemaker<br>Prior Stroke<br>Prior Sternotomy<br>Elixhauser comorbidities Index	**Clinical Presentation**<br>STEMI (or AMI for CABG)<br>NSTEMI (or AMI for CABG)<br>UA/SIHD<br><br>**PCI Characteristics**<br>Multivessel PCI<br>IVUS/FFR Use<br>Chronic Total Occlusion<br>Bare Metal Stent Use<br>Cardiogenic Shock<br>Mechanical Circulatory Support<br><br>**CABG Characteristics**<br>Isolated CABG<br>1-2 Vessel CABG<br>≥ 3 Vessel CABG<br>Off Pump CABG<br>Double IMAs<br>Any Arterial Conduit<br>Cardiogenic Shock<br>Intra-aortic Balloon Pump Use

eTable 2. Variables Included in the Regression Models

CABG; coronary artery bypass grafting, AMI; acute myocardial infarction, PCI; percutaneous coronary intervention, STEMI; ST-elevation myocardial infarction, NSTEMI; non-ST elevation myocardial infarction, UA; unstable angina, SIHD; stable ischemic heart disease, ICD; internal cardioverter defibrillator, IVUS; intravascular ultrasound, FFR; functional flow reserve, IMA; Internal mammary artery

© 2020 Alkhouli M et al. *JAMA Network Open.*

Year	2003	2004	2005	2006	2007	2008	2009	2010	2011	2012	2013	2014	2015	2016
**PCI**	777780	770217	779659	878519	698705	731981	668358	538277	540043	536125	500555	466820	469680	440505
**CABG**	337444	298811	270436	294295	241792	252252	258119	207215	204914	202950	199935	201625	203115	201840
**US Adults**	212622 000	214700 000	217374 000	219849 000	222722 000	224703 000	226973 000	229240 000	231195 000	234719 000	236929 000	239448 000	242247 000	244807 000
**PCI per 100,000***	365.8	358.7	358.7	399.6	313.7	325.8	294.5	234.8	233.6	228.4	211.3	195.0	193.9	179.9
**CABG per 100,000***	158.7	139.2	124.4	133.9	108.6	112.3	113.7	90.4	88.6	86.5	84.4	84.2	83.8	82.4

eTable 3. Temporal Trends in the Volume of PCI and CABG (Total Numbers and Rates Per 100.000 US Adults)

PCI; percutaneous coronary intervention, CABG; coronary artery bypass grafting, US; United States

© 2020 Alkhouli M et al. *JAMA Network Open.*

Post PCI Mortality	2003	2004	2005	2006	2007	2008	2009	2010	2011	2012	2013	2014	2015	2016	ARR Per Year	95% CI		P Trend
**PCI for STEMI**																		
Unadjusted	4.9%	5.0%	5.3%	5.2%	5.7%	6.0%	5.8%	5.8%	6.3%	6.3%	6.1%	6.8%	6.2%	5.5%	1.014	1.012	1.016	<0.001
Adjusted	4.9%	5.2%	5.4%	5.4%	6.1%	5.9%	5.6%	5.4%	5.9%	5.7%	5.6%	6.0%	5.5%	5.3%	1.009	1.007	1.012	<0.001
**PCI for NSTEMI**																		
Unadjusted	1.6%	1.8%	1.8%	1.7%	1.7%	1.8%	1.8%	1.7%	1.6%	1.8%	1.8%	1.8%	2.0%	1.9%	1.007	1.004	1.01	<0.001
Adjusted	1.6%	1.7%	1.7%	1.7%	1.6%	1.6%	1.6%	1.6%	1.3%	1.5%	1.5%	1.5%	1.5%	1.6%	1.000	0.998	1.002	0.18
**PCI for US/SIHD**																		
Unadjusted	0.8%	0.8%	0.7%	0.7%	0.8%	1.0%	1.1%	1.2%	1.4%	1.6%	1.8%	2.2%	1.9%	1.0%	1.097	1.095	1.099	<0.001
Adjusted	0.8%	0.8%	0.8%	0.8%	0.8%	1.0%	1.0%	1.1%	1.1%	1.2%	1.3%	1.4%	1.3%	1.0%	1.019	1.018	1.021	<0.001

eTable 4. Temporal Trends of Crude (unadjusted) and Risk-Adjusted In-Hospital Mortality Rates Following PCI

PCI; percutaneous coronary intervention, STEMI; ST-elevation myocardial infarction, NSTEMI; non-ST elevation myocardial infarction, UA; unstable angina, SIHD; stable ischemic heart disease, ARR; adjusted rate ratio, C.I.; confidence interval

© 2020 Alkhouli M et al. *JAMA Network Open.*

CABG Mortality	2003	2004	2005	2006	2007	2008	2009	2010	2011	2012	2013	2014	2015	2016	ARR Per Year	95% CI		P Trend
**CABG for AMI**																		
Unadjusted	5.6%	5.5%	5.2%	4.4%	4.6%	4.5%	4.4%	3.6%	3.8%	3.5%	3.7%	3.5%	3.9%	3.6%	0.962	0.959	0.965	<0.001
Adjusted	5.6%	6.0%	5.4%	4.6%	4.7%	4.5%	4.4%	3.5%	3.4%	3.5%	4.0%	3.5%	3.9%	3.4%	0.981	0.978	0.984	<0.001
**CABG for UA/SIHD**																		
Unadjusted	2.8%	2.5%	2.5%	2.5%	2.3%	2.5%	2.1%	2.0%	2.2%	2.1%	2.0%	2.0%	2.1%	1.7%	0.972	0.97	0.974	<0.001
Adjusted	2.8%	2.6%	2.5%	2.4%	2.2%	2.3%	1.9%	1.8%	1.9%	1.8%	1.7%	1.7%	1.8%	1.7%	0.987	0.985	0.989	<0.001

eTable 5. Temporal Trends of Crude (unadjusted) and Risk-Adjusted In-Hospital Mortality Rates Following CABG

CABG; coronary artery bypass grafting, AMI; acute myocardial infarction, C.I.; confidence interval

© 2020 Alkhouli M et al. *JAMA Network Open.*

CABG Mortality	2003	2004	2005	2006	2007	2008	2009	2010	2011	2012	2013	2014	2015	2016	ARR Per Year	95% CI		P Trend
**CABG for AMI**																		
Unadjusted	4.8%	4.8%	4.4%	3.8%	3.9%	4.0%	3.7%	3.0%	3.3%	3.1%	3.2%	3.0%	3.4%	3.2%	0.963	0.961	0.966	<0.001
Adjusted	4.8%	5.2%	4.6%	3.9%	4.0%	4.0%	3.8%	3.0%	2.9%	3.1%	3.4%	2.9%	3.3%	3.0%	0.981	0.977	0.984	<0.001
**CABG for UA/SIHD**																		
Unadjusted	2.1%	1.9%	1.8%	1.7%	1.6%	1.7%	1.4%	1.4%	1.5%	1.4%	1.4%	1.4%	1.5%	1.2%	0.965	0.963	0.968	<0.001
Adjusted	2.1%	2.1%	1.9%	1.8%	1.6%	1.6%	1.4%	1.3%	1.4%	1.4%	1.3%	1.2%	1.3%	1.2%	0.988	0.986	0.99	<0.001

eTable 6. Temporal Trends of Crude (unadjusted) and Risk-Adjusted In-Hospital Mortality Following Isolated CABG

CABG; coronary artery bypass grafting, AMI; acute myocardial infarction, C.I.; confidence interval, Ref; reference

© 2020 Alkhouli M et al. *JAMA Network Open.*

Patients Undergoing PCI and CABG During the Same Admission														
**Year**	**2003**	**2004**	**2005**	**2006**	**2007**	**2008**	**2009**	**2010**	**2011**	**2012**	**2013**	**2014**	**2015**	**2016**
Number of patients	9504	8773	8446	9430	8659	8904	9045	7395	6976	6995	6970	6560	6495	5735
Proportion of all PCIs	0.9%	0.8%	0.8%	0.8%	0.9%	0.9%	1.0%	1.0%	0.9%	1.0%	1.0%	1.0%	1.0%	0.9%
In-Hospital Mortality	6.5%	5.9%	6.4%	5.4%	4.9%	5.9%	5.2%	4.3%	6.4%	6.3%	5.7%	6.3%	6.8%	5.9%
**Patients Undergoing Either PCI or CABG During the Same Admission**														
In-Hospital Mortality (PCI group)	1.2%	1.2%	1.2%	1.2%	1.4%	1.5%	1.6%	1.8%	1.9%	2.1%	2.2%	2.6%	2.6%	2.7%
In-Hospital Mortality (CABG group)	3.3%	3.1%	3.0%	2.8%	2.8%	2.9%	2.6%	2.4%	2.6%	2.4%	2.4%	2.4%	2.6%	2.3%

eTable 7. Temporal Trends in Unadjusted In-Hospital Mortality Among Patients Undergoing PCI, CABG, or PCI and CABG During the Same Hospitalization

PCI; percutaneous coronary intervention, CABG; coronary artery bypass grafting

© 2020 Alkhouli M et al. *JAMA Network Open.*

Year	2003	2004	2005	2006	2007	2008	2009	2010	2011	2012	2013	2014	2015	2016
**CABG**														
AMI	12.27 (10.2)	12.53 (9.98)	12.6 (9.97)	12.15 (9.54)	12.63 (10.73)	12.43 (9.33)	12.41 (9.84)	11.82 (8.59)	12.22 (8.32)	12.16 (8.77)	11.96 (8.53)	12.11 (9.24)	11.9 (8.52)	11.82 (8.05)
UA/SIHD	9.3 (8.14)	9.35 (8.23)	9.51 (8.09)	9.4 (8.49)	9.5 (8.18)	9.33 (7.69)	9.17 (7.58)	8.89 (7.19)	9.3 (7.35)	9.17 (7.02)	9.02 (6.65)	8.95 (6.46)	8.98 (6.77)	8.85 (6.79)
**Isolated CABG**														
AMI	11.83 (9.72)	12.05 (9.42)	12 (9.17)	11.72 (9.07)	12.13 (10.14)	11.87 (8.7)	11.89 (9.3)	11.44 (8.21)	11.76 (7.82)	11.81 (8.54)	11.55 (7.92)	11.71 (8.4)	11.55 (8.08)	11.51 (7.68)
UA/SIHD	8.74 (7.1)	8.8 (7.35)	8.89 (6.98)	8.77 (7.39)	8.85 (7.03)	8.7 (6.76)	8.55 (6.38)	8.36 (6.31)	8.67 (6.46)	8.58 (6.21)	8.51 (5.97)	8.43 (5.71)	8.51 (5.93)	8.42 (6.03)
**PCI**														
STEMI	4.91 (5.45)	4.87 (4.98)	4.8 (5.14)	4.72 (5.72)	4.65 (5.12)	4.64 (5.49)	4.56 (5.14)	4.47 (4.85)	4.45 (5.35)	4.41 (5.4)	4.28 (5.43)	4.18 (5.13)	4.01 (5.26)	3.77 (5.16)
NSTEMI	4.46 (4.99)	4.47 (5.52)	4.36 (4.91)	4.24 (4.92)	4.13 (4.66)	4.18 (4.86)	4.23 (4.77)	4.23 (4.73)	4.17 (4.82)	4.02 (4.78)	4.02 (4.88)	4.02 (4.74)	4.03 (4.64)	4.04 (4.92)
UA/SIHD	2.64 (3.51)	2.58 (3.43)	2.47 (3.29)	2.38 (3.21)	2.47 (3.51)	2.52 (3.42)	2.72 (3.59)	2.98 (3.93)	3.02 (4.26)	3.05 (4.17)	3.26 (4.95)	3.36 (5.34)	3.35 (5.16)	3.2 (5.06)

eTable 8. Temporal Trends of Length of Stay Following PCI and CABG

CABG; coronary artery bypass grafting, AMI; acute myocardial infarction, PCI; percutaneous coronary intervention, STEMI; ST-elevation myocardial infarction, NSTEMI; non-ST elevation myocardial infarction, UA; unstable angina, SIHD; stable ischemic heart disease
Values are presented as mean (standard deviation)

© 2020 Alkhouli M et al. *JAMA Network Open.*
